# Elevated plasma protein carbonylation increases the risk of ischemic cerebrovascular events in patients with atrial fibrillation: association with a prothrombotic state

**DOI:** 10.1007/s11239-024-03003-z

**Published:** 2024-07-04

**Authors:** Karol Nowak, Michal Zabczyk, Joanna Natorska, Jaroslaw Zalewski, Anetta Undas

**Affiliations:** 1 Department of Thromboembolic Disorders, Institute of Cardiology, Jagiellonian University Medical College, Pradnicka 80 St, 31-202 Kraków, Poland; 2 Department of Coronary Artery Disease and Heart Failure, John Paul II Hospital, Pradnicka 80 St, 31-202 Kraków, Poland; 3 Krakow Centre for Medical Research and Technologies, John Paul II Hospital, Pradnicka 80 St, 31-202 Kraków, Poland; 4 Department of Coronary Artery Disease and Heart Failure, Institute of Cardiology, Jagiellonian University Medical College, Pradnicka 80 St, 31-202 Kraków, Poland

**Keywords:** Atrial fibrillation, Protein carbonylation, Stroke, Fibrin clot, Endogenous thrombin potential

## Abstract

**Introduction:**

Plasma protein carbonylation that reflects oxidative stress has been demonstrated to be associated with the prothrombotic fibrin clot phenotype. However, the role of protein carbonyls (PC) in predicting ischemic stroke in atrial fibrillation (AF) is largely unknown. This study aimed to investigate whether PC increase the risk of stroke in anticoagulated AF patients during follow-up.

**Methods:**

In 243 AF patients on anticoagulation (median age 69 years; median CHA_2_DS_2_-VASc of 4), we measured plasma PC using the assay by Becatti, along with plasma clot permeability (K_s_), clot lysis time (CLT), thrombin generation, and fibrinolytic proteins, including plasminogen activator inhibitor type 1 (PAI-1) and thrombin activatable fibrinolysis inhibitor (TAFI). Ischemic stroke, major bleeding, and mortality were recorded during a median follow-up of 53 months.

**Results:**

Plasma PC levels (median, 3.16 [2.54–3.99] nM/mg protein) at baseline showed positive associations with age (*P* < 0.001), CHA_2_DS_2_-VASc (*P* = 0.003), and N-terminal B-type natriuretic peptide (*P* = 0.001), but not with type of AF or comorbidities except for heart failure (*P* = 0.007). PC levels were correlated with CLT (r = 0.342, *P* < 0.001), endogenous thrombin potential (r = 0.217, *P* = 0.001) and weakly with Ks (r = -0.145, *P* = 0.024), but not with fibrinogen, PAI-1, or TAFI levels. Stroke was recorded in 20 patients (1.9%/year), who had at baseline 36% higher PC levels (*P* < 0.001). Elevated PC (*P* = 0.003) at baseline were independently associated with stroke risk.

**Conclusion:**

Our findings suggest that in patients with AF enhanced protein carbonylation is associated with increased “residual” risk of stroke despite anticoagulation, which is at least in part due to unfavorably altered fibrin clot phenotype.

**Graphical Abstract:**

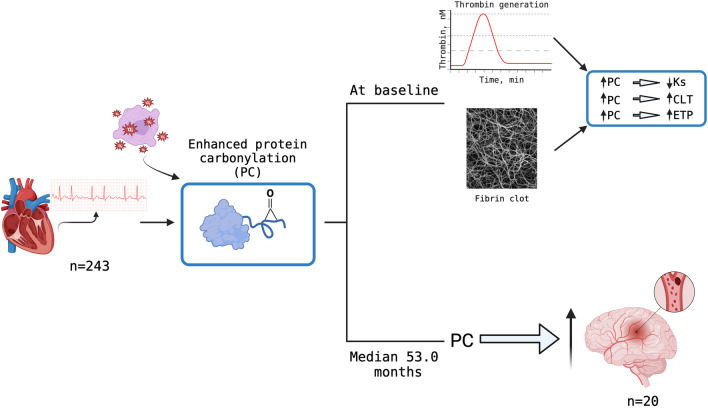

**Supplementary Information:**

The online version contains supplementary material available at 10.1007/s11239-024-03003-z.

## Introduction

Oxidative stress, the imbalance between reactive oxygen species (ROS) generation and antioxidant capacity [1], leads to a variety of oxidative protein modifications including carbonylation, which is a direct, metal catalyzed reaction with amino-acid side chains generating the glutamic semialdehyde from arginine and proline, and aminoadipic semialdehyde from lysine [2]. Since the reaction is irreversible, elevated protein carbonyls (PC) levels are considered an early biomarker of exposure on ROS [2]. Enhanced protein carbonylation has been observed in arterial and venous thromboembolism (VTE) [3–5]. Fibrinogen, clotting factors (F) V, VIII, X, XIII, and fibrinolysis proteins including tissue plasminogen activator (tPA) are sensitive to oxidation [6]. It has been shown in vitro that oxidative posttranslational fibrinogen modifications impair kinetics of fibrin clot formation and alter fibrin structure and biomechanical function [7]. Carbonylation of fibrinogen molecules has been suggested to increase thromboembolic risk, along with clot resistance to plasmin-induced lysis [5]. A recent study has demonstrated that in patients with acute ischemic stroke, PC were associated with the severity of neurological deficits and post stroke mortality, along with more compact fibrin clot formation and impaired clot lysability [4].

Compelling evidence indicates the role of enhanced oxidative stress in the pathophysiology of atrial fibrillation (AF) and heart failure (HF) [8, 9]. ROS exposure causes myocardial cell dysfunction which activates angiotensin II, endothelin-1, and tumor necrosis factor-α leading to further cardiac injury, fibrosis, and necrosis, contributing to AF and HF [8, 9]. AF increases risk of ischemic strokes, systemic embolism, and transient ischemic attacks (TIAs) [10], also in HF patients [11]. Multiple mechanisms underlying thromboembolic risk have been reported in AF [12]. The contribution of enhanced oxidative stress has been highlighted recently, as evidenced by elevated levels of 8-isoprostane or myeloperoxidase [13, 14]. Higher PC levels in atrial tissue samples obtained from patients with AF during cardiac surgery have been reported [15]. To our knowledge there have been no reports evaluating circulating PC levels in AF and their prognostic value. Therefore, we investigated whether plasma PC levels affect the risk of cardiovascular events in in a cohort of anticoagulated AF patients.

## Material and methods

We enrolled 243 consecutive adult patients with documented non-valvular AF according to European Society of Cardiology (ESC) Guidelines [16], who were admitted to John Paul II Hospital in Cracow, Poland, between June 2014 and July 2016. The study population was presented in detail previously [14]. Patients with acute myocardial infarction (MI), VTE within last 12 months, end-stage chronic kidney disease, severe liver injury, active cancer, pregnancy, symptoms of acute infection and C-reactive protein (CRP) > 10 mg/L were excluded. The risk of stroke was evaluated by CHA_2_DS_2_-VASc score [16]. The AF subtypes were assessed according to the 2012 ESC guidelines [16]. The diagnosis of HF and prior MI was established based on ESC guidelines [17, 18]. Reduced left ventricular ejection fraction (LVEF) on transthoracic echocardiography was defined as ≤ 40% [17]. Ischemic stroke was recognized based on typical symptoms confirmed on computed tomography and magnetic resonance imaging [19]. Fasting glycemia ≥ 7 mmol/L on 2 separate occasions or the hypoglycemic therapy were the criteria for diabetes mellitus (DM). The Local Ethic Committee of Jagiellonian University approved the study protocol, and the study was conducted in accordance with the Declaration of Helsinki. All participants gave their written informed consent. The study adhered to the STROBE reporting guidelines.

### Laboratory investigations

Regarding patients on direct oral anticoagulants (DOACs), the fasting blood was collected 24–28 h since the administration of the last dose of rivaroxaban and 12–18 h since the administration of apixaban or dabigatran. Patients on vitamin K antagonists (VKA) had blood drawn at least 24 h after the last dose of low-molecular-weight heparin administered as bridging therapy. Blood was drawn between 8 and 10 AM from the antecubital vein to tubes containing (9:1) 3.2% trisodium citrate which were centrifuged at 2500 g at 20 °C for 20 min whereas serum tubes were centrifuged at 1600 g at 4 °C for 10 min. Aliquots were stored at -80 °C. Routine laboratory investigations, including N-terminal B-type natriuretic peptide (NT-proBNP) were assessed by routine hospital techniques. Plasminogen activator inhibitor-1 (PAI-1) antigen and thrombin activatable fibrinolysis inhibitor (TAFI) activity (both Hyphen-Biomed, Neuville-Sur-Oise, France) were assayed using ELISA kits. Fibrinogen was determined by the Clauss method. The quantitative determination of the plasminogen and α2-antiplasmin activity were assessed in citrated plasma by the synthetic chromogenic substrate method with the use of a commercial calibration standard and values were expressed as a percentage of normal (both STA-Stachrom, Diagnostica Stago, Asnières-sur-Seine, France). In this assay, plasma was incubated with the streptokinase reagent which consist of streptokinase, human albumin, and buffer in the presence of fibrinogen. Then the plasminogen activity was quantified by the plasminogen-streptokinase complexes action on the synthetic chromogenic substrate, as the amount of p-nitroaniline release, measured at 405 nm. The Von Willebrand factor (vWF) antigen was evaluated by latex immunoassay (Diagnostica Stago, Asnières-sur-Seine, France). All results have been presented according to the manufacturer’s instructions.

### Carbonylation

Carbonyl contents were assessed using the reaction of 2,4-dinitrophenylhydrazine (DNPH) with PC, which leads to the formation of a Schiff base, and subsequently, the corresponding hydrazone which could be analyzed spectrophotometrically, according to Becatti et al. [5]. Briefly, 100 μL of plasma was incubated with 400 μL of DNPH and then precipitated with trichloracetic acid. Subsequently, the pellet was washed several times with a 1:1 mixture of ethanol/ethyl acetate and resuspended in 500 μL of guanidine hydrochloride. Absorbance at 370 nm was measured using spectrophotometer (Tecan, Sunrise). Carbonyls contents were evaluated by using a molar extinction coefficient of 22,000 mol/L^−1^ cm^−1^ and expressed as nmol/mL of PC per 1 mg of protein. The in-house reference range for healthy subjects is 0.54–2.03 nmol/mg [3]. The intra-assay variability was 5.9%, whereas inter-assay variability equaled 7.8%.

### Endogenous thrombin potential

Calibrated automated thrombogram was assessed as described [20]_,_ and performed according to manufacturer’s instructions (Thrombinoscope BV, Maastricht, Netherlands). Briefly, the assay was performed in a 96-well plate fluorometer (Ascent Reader, Thermolabsystems OY, Helsinki, Finland). To 80 μL platelet-poor plasma 20 μL of (TF)-based activator (PPP Reagent; final TF concentration, 5 pM) and FluCa solution (both Diagnostica Stago) were added. Fluorescence readings began immediately (at 390 nm excitation and 460 nm emission wavelengths, at 37 °C) was followed over a 60 min period. Each plasma sample was analyzed in duplicate. Endogenous thrombin potential (ETP), calculated as the area under the curve of thrombin formed in time, was used to measure thrombin generation capacity. Inter-assay coefficients of variation were < 7%.

### Fibrin clot assessment

Fibrin clot permeability and fibrinolysis capacity determined as clot lysis time (CLT) were assessed as described [21]. Briefly, to assess clot permeability CaCl_2_ (20 mM) and human thrombin (1 U/mL; Sigma-Aldrich, St Louis, USA) were mixed with citrated plasma. The permeation coefficient (Ks) reflecting the average size of pores formed in the fibrin network. Ks was calculated as follows: Ks = Q × L × η / t × A × Δp. Q is the flow rate in percolating time (t), L is the length of a fibrin gel, η is the viscosity of liquid, A is the cross-sectional area, and Δp is a differential pressure. The interassay and interassay coefficients of variation were < 7%. CLT was defined as the time from the midpoint of the clear-to-maximum-turbid transition, representing clot formation, to the midpoint of the maximum-turbid-to-clear transition representing clot lysis. 12 μmol/L phospholipid vesicles, 15 mmol/L CaCl_2_, 0.6 pmol/L tissue factor (TF) (Innovin, Siemens) and 60 ng/mL recombinant tissue plasminogen activator (tPA) (Boehringer Ingelheim, Ingelheim, Germany) were mixed with citrated plasma to evaluate CLT. The turbidity was measured at 405 nm. The intra-assay variability was 8%.

### Follow-up

The long-term outcomes were assessed by telephone or clinical visit for at least twice a year. The primary endpoint was ischemic stroke defined as shown above. The secondary endpoints were death and major bleeding defined according to the ISTH bleeding tool assessment [22].

### Statistical analysis

The study was powered to have a 90% chance of detecting a 20% difference with 20% standard deviation in PC using a *P* value of 0.05 between AF patients with and without stroke, assuming stroke event rate for 8–9%. Based on the data in the published articles [4], to demonstrate such a difference or greater, 14 patients with and 138 without stroke were required in each group. For a *P* value of 0.01, 20 and 196 patients were required in respective groups.

Statistical analyses were performed using the SPSS Statistics software (Version 29.0.0.0, IBM Corp., Armonk, NY, USA). Continuous variables were expressed as a median (interquartile range [IQR]), whereas categorical variables were shown as a number (percentage). Normal distribution was assessed by the Shapiro–Wilk test. Intergroup differences were evaluated by Student’s t-test when normally distributed or by the Mann–Whitney U test for non-normally distributed variables. Analysis of variance followed by a post hoc Bonferroni test was used to compare differences of single measurements in more than two groups with normally distributed data whereas non-normally distributed data were analyzed by Kruskal–Wallis test and differences between groups were identified using a test for multiple comparisons of mean ranks. Categorical variables were compared by Fisher’s exact test. The Pearson or Spearman rank correlation coefficients were calculated to test the association between two variables having a normal or non-normal distribution, respectively. All independent variables potentially associated with both the exposure and outcome, and which lacked significant correlation with other independent variables, were included in the Cox proportional hazard regression to determine predictors of stroke as well as major bleeding or included in the linear multivariable analysis to find parameters independently associated with fibrin clot properties. A two-tailed *P* < 0.05 was considered statistically significant.

## Results

We assessed 243 patients (44% women) as shown in Table 1. The median time from AF diagnosis to inclusion was 6 (4.0–8.2) years and 83.5% of subjects had high thromboembolic risk (CHA_2_DS_2_-VASc score, median 4.0 [3.0–5.0] points). Permanent AF (39.1%) was the most prevalent manifestation. Most patients were treated with DOACs (*n* = 171, 70.3%; Table 1), however 7 subjects stopped anticoagulation during observation.

**Table 1:** The baseline characteristics of patients with and without cerebrovascular ischemic events in follow-up

Variable	All patients, *N* = 243	With stroke, *n* = 20	Without stroke, *n* = 219	*P*-value
Age, years	69 (64–75)	68.5 (62–77.3)	69 (64–75)	0.72
Women, n (%)	107 (44.0)	11 (55.0)	96 (43.0)	0.35
BMI, kg/m^2^	28.1 (25.4–31.6)	26.6 (23.8–30.7)	28.3 (25.5–31.7)	0.17
Current smoking, n (%)	87 (35.8)	8 (40.0)	79 (35.4)	0.80
Data on AF				
Paroxysmal AF, n (%)	77 (31.7)	4 (20.0)	73 (32.7)	0.31
Persistent AF, n (%)	71 (29.2)	9 (45.0)	62 (27.8)	0.12
Permanent AF, n (%)	95 (39.1)	7 (35.0)	88 (39.5)	0.81
Time from AF diagnosis, years	6.0 (4.0–8.2)	6.0 (5.0–8.0)	6.0 (4.0–9.0)	0.45
CHA_2_DS_2_-VASc score, points	4.0 (3.0–5.0)	4.5 (3.3–5.0)	4.0 (3.0–5.0)	0.058
Comorbidities				
Hypertension, n (%)	187 (77.0)	16 (80.0)	171 (76.7)	0.99
Diabetes mellitus, n (%)	71 (29.2)	9 (45.0)	62 (27.8)	0.09
Dyslipidemia, n (%)	214 (88.1)	17 (85.0)	197 (88.3)	0.72
Prior MI, n (%)	71 (29.2)	6 (30.0)	65 (29.1)	0.99
Prior stroke, n (%)	58 (23.9)	7 (35.0)	51 (22.9)	0.27
Left ventricle ejection fraction, (%)	49 (42–55)	45 (38.3–53)	50 (42–55)	0.041
Medications				
ASA, n (%)	105 (43.2)	8 (40.0)	97 (43.5)	0.81
Statins, n (%)	157 (64.6)	14 (70.0)	143 (64.1)	0.80
Rivaroxaban, n (%)	80 (32.9)	6 (30.0)	74 (33.2)	0.99
Dabigatran, n (%)	56 (23.0)	4 (20.0)	52 (23.3)	0.99
Apixaban, n (%)	35 (14.4)	3 (15.0)	32 (14.3)	0.99
Warfarin, n (%)	61 (25.1)	7 (35.0)	54 (24.2)	0.23
The laboratory results				
Hemoglobin, g/dl	13.9 (13.2–14.9)	13.5 (13.0–14.7)	13.9 (13.0–14.9)	0.40
White blood cell count, × 10^3^/µL	6.7 (5.5–7.5)	6.9 (5.4–7.9)	6.6 (5.5–7.5)	0.87
Platelets, × 10^3^/µL	210 (176–251)	212 (179–290)	210 (175–249)	0.11
Fasting glucose, mmol/L	5.4 (4.9- 6.2)	5.5 (5.0–6.2)	5.4 (4.9–6.2)	0.81
Creatinine, µmol/L	82 (71.8–98.2)	98 (77.5–122.7)	82 (71.2–97)	0.01
Total cholesterol, mmol/L	4.5 (3.8–5.6)	4.3 (3.9–5.1)	4.5 (3.8–5.6)	0.67
LDL-cholesterol mmol/L	2.59 (2.03–3.40)	2.31 (1.99–2.85)	2.60 (2.04–3.41)	0.26
HDL-cholesterol, mmol/L	1.32 (1.08–1.90)	1.26 (1.12–1.73)	1.32 (1.08–1.62)	0.71
Triglycerides, mmol/L	1.28 (0.9–1.8)	1.23 (0.8–1.7)	1.28 (0.9–1.8)	0.41
INR	1.01 (0.95–1.07)	1.02 (0.98–1.07)	1.01 (0.94–1.07)	0.62
APPT, s	29.0 (26.2–30.9)	29.6 (26.0–31.8)	28.9 (26.2–30.7)	0.39
CRP, mg/L	1.8 (1.1–3.2)	1.7 (1.0–2.3)	1.8 (1.2–3.3)	0.45
NT-proBNP, pg/mL	748 (391–1493)	2635 (1209–3263)	699 (348–1328)	< 0.001
Ks, × 10^−9^ cm2	6.5 (6.0–7.2)	6.2 (5.5–6.7)	6.6 (6.0–7.4)	0.010
CLT, min	94 (80–108)	108 (83–130)	94 (80–105)	< 0.001
ETP, nM × min	1502 (1427–1638)	1660 (1505–1725)	1494 (1423–1633)	< 0.001
Fibrinogen, g/L	3.2 (2.5–3.9)	3.3 (2.5–4.1)	3.2 (2.5- 3.9)	0.44
vWF:Ag, %	208 (174–243)	241 (204–287)	202 (171–239)	< 0.001
TAFI:Ag, %	100 (89–110)	95 (87–110)	100 (90–110)	0.41
Plasminogen, %	105 (95–115)	104 (97–110)	105 (94–117)	0.88
Antiplasmin, %	107 (96–117)	111 (103–120)	106 (96–117)	0.19
PAI-1:Ag, ng/mL	14.0 (10.8–18.7)	16.3 (8.9–21)	13.9 (10.8–18.6)	0.08
Protein carbonylation, nM/mg	3.16 (2.54–3.99)	4.20 (3.62 -4.83)	3.08 (2.51–3.81)	< 0.001

Values are shown as numbers (percentage) or median (interquartile range) as appropriate. *AF* – atrial fibrillation, *ASA* – acetylsalicylic acid, *BMI* – body mass index, *CRP* – c-reactive protein, *CLT* – clot lysis time, *ETP* – endogenous thrombin potential, *HDL-cholesterol* – high-density lipoprotein cholesterol, *LDL-cholesterol* – low-density lipoprotein cholesterol, *MI* – myocardial infarction, *NT-proBNP *– N-terminal B-type natriuretic peptide, *PAI-1:Ag *– plasminogen activator inhibitor type 1 antigen, *TAFI:Ag *– thrombin activatable fibrinolysis inhibitor antigen, *vWF:Ag* – von Willebrand factor antigen

**Fig. 1:**
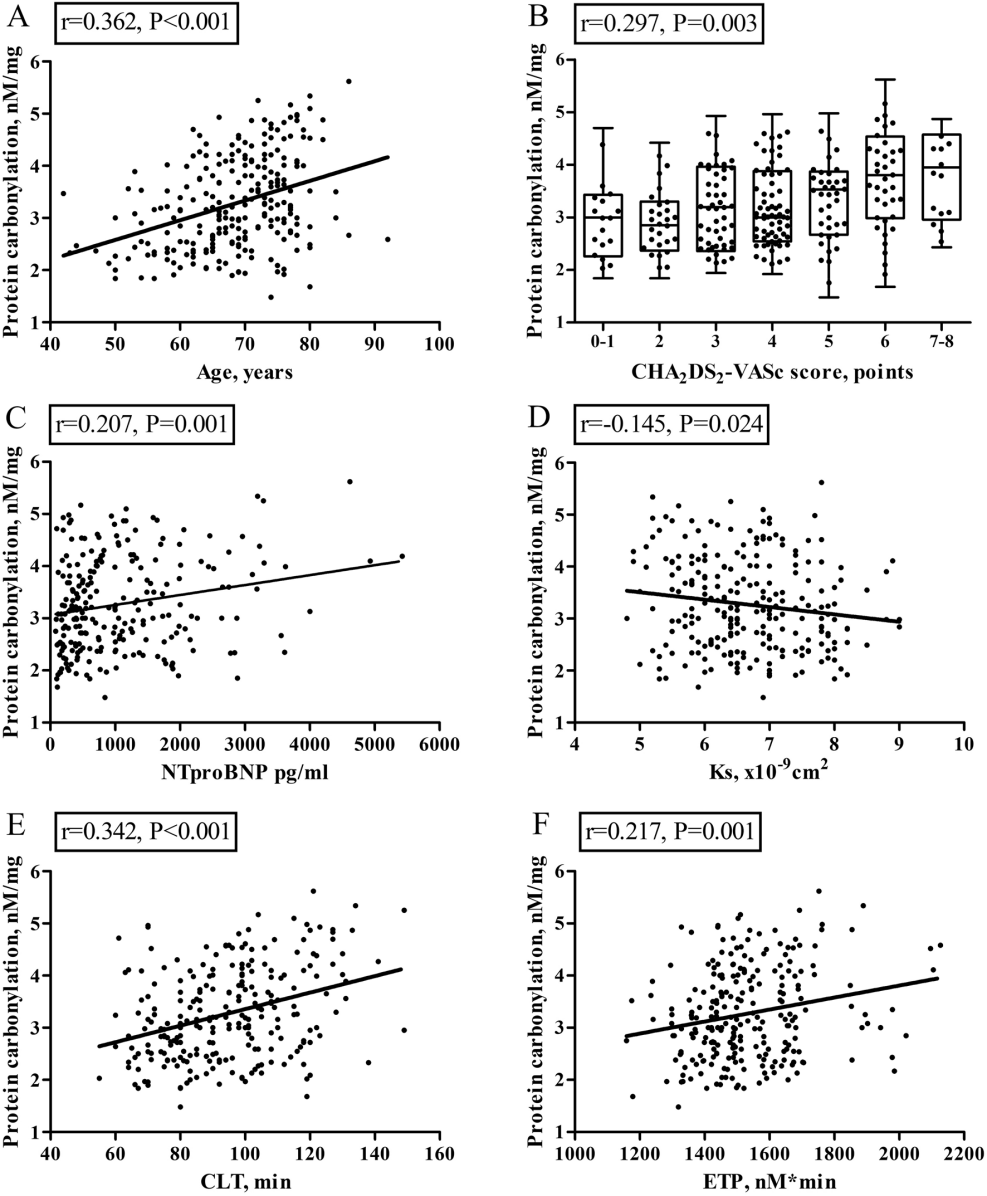
The significant correlation between plasma protein carbonylation, CHA_2_DS_2_-VASc and laboratory tests

The median PC level was 3.16 (2.54–3.99) nM/mg and the vast majority of patients (*n* = 227, 93.4%) had this level above upper limit of the reference range. There were positive associations of PC with age (r = 0.362, *P* < 0.001, Fig. 1A), CHA_2_DS_2_-VASc (r = 0.297, *P* = 0.003, Fig. 1B), and NT-proBNP level (r = 0.207, *P* = 0.001, Fig. 1C) but not with other demographics, comorbidities except for HF (r = 0.174, *P* = 0.007) and medications. After adjustment for age and HF diagnosis, CHA_2_DS_2_-VASc was no longer associated with PC level (r = 0.123, *P* = 0.08). By multivariable linear regression model age and NT-proBNP levels were independent predictors of PC (*P* < 0.001 for both).

There were no differences in PC levels related to the type of anticoagulants (*P* = 0.16). In patients on VKA or DOACs, median PC levels were 3.04 (2.53–3.84) and 3.20 (2.55–3.99) nM/mg, respectively. PC correlated with fibrin clot properties, namely Ks (r = -0.145, *P* = 0.024, Fig. 1D), and CLT (r = 0.342, *P* < 0.001, Fig. 1E), as well as with thrombin generation, reflected by ETP (r = 0.217, *P* = 0.001, Fig. 1F) and weakly with plasminogen activity (r = -0.182, *P* = 0.004), but not with fibrinogen, CRP, TAFI, and PAI-1 levels (all *P* > 0.05). After adjustment for age, higher plasma NT-proBNP (ß 0.276, 95% CI 0.158–498) and PC levels (ß 0.331, 95% CI 0.205–0.456) were independently associated with longer CLT (R2 = 0.211, *P* < 0.001).

Patients with concomitant HF (*n* = 57, 23.5%) with reduced LVEF (HFrEF) (Online Resource 1) had higher PC levels by 23% (*P* = 0.005) as compared to those free of this disease, while other comorbidities had no impact of this variable. The former group comprised more current smokers (*P* = 0.017), subjects with previous MI (*P* = 0.002), who had higher CHA_2_DS_2_-VASc (*P* < 0.001) and slightly higher white blood cell count (*P* = 0.041) and creatinine (*P* < 0.001). Those patients had slightly lower PAI-1 (*P* = 0.024) and TAFI (*P* = 0.040) antigens, respectively. In AF patients with HFrEF there were positive correlations of PC levels with CLT (r = 0.317, *P* < 0.001) and ETP (r = 0.208, *P* = 0.001) along with a weak, inverse association between PC levels and Ks (r = -0.146, *P* = 0.023) as well as plasminogen (r = -0.162, *P* = 0.011). After adjustment for age and diagnosis of HFrEF, associations of PC in the whole cohort with Ks (r = -0.154, *P* = 0.026), CLT (r = 0.398, *P* < 0.001), and ETP (r = 0.306, *P* < 0.001) remained significant.

### Long-term follow-up

The median follow-up time was 53.0 (47.0–56.5) months. Four patients were lost to follow-up, therefore in the final analysis 239 patients were included.

We recorded 20 ischemic strokes events (8.4%, 1.9%/year), 23 (9.6%, 2.2%/year) major bleedings, and 20 (8.4%, 1.9%/year) deaths during follow-up. No systemic embolism was reported. Patients who experienced stroke were similar to the remainder except for lower LVEF by 11% (*P* = 0.041) at baseline (Table 1) In the former group higher creatinine by 13% (*P* = 0.013), NT-proBNP by 265% (*P* < 0.001), and vWF by 18% (*P* = 0.01) were noticed. Patients with cerebrovascular events were characterized by 8% higher ETP (*P* < 0.001), 15% longer CLT (*P* = 0.015), and 8% lower Ks (*P* = 0.011) at baseline.

Ischemic strokes were observed in individuals with 36% higher PC at baseline (*P* < 0.001) as compared with subjects free of such events (Table 1). By Cox proportional regression analysis, age (*P* = 0.037), higher NT-proBNP (*P* < 0.001), and PC (*P* = 0.003) were associated with an increased stroke risk (Table 2).

**Table 2:** The independent predictors of long-term outcomes

The independent predictors of stroke (Chi^2^ = 46.8, df = 6, *P* < 0.0001)								
Independent variables	Univariate model				Multivariate model			
	P-value	HR	95% CI for HR		P-value	HR	95% CI for HR	
Age, per 1 year	0.636	1.013	0.959	1.069	0.037	0.928	0.866	0.995
Sex, female vs male	0.290	1.609	0.666	3.882	0.144	2.064	0.782	5.452
Creatinine, per 1 umol/l	0.010	1.017	1.004	1.030	0.181	1.014	0.994	1.034
NT-proBNP, per 100 pg/ml	< 0.001	1.096	1.065	1.128	< 0.001	1.070	1.032	1.109
CLT, per 1 min	0.002	1.036	1.013	1.059	0.843	0.997	0.972	1.024
Protein carbonylation, per 1 nM/mg	< 0.001	3.457	2.005	5.958	0.003	3.290	1.512	7.159
The independent predictors of major bleeding (Chi^2^ = 28.1, df = 5, *P* < 0.0001)								
Independent variables	Univariate model				Multivariate model			
	P-value	HR	95% CI for HR		P-value	HR	95% CI for HR	
Age, per 1 year	0.649	1.013	0.960	1.070	0.046	0.925	0.857	0.998
Sex, female vs male	0.790	1.119	0.490	2.553	0.164	0.507	0.194	1.321
CHA2DS2-VASc, per 1 point	0.004	1.440	1.119	1.853	0.002	1.797	1.234	2.617
Ks, per 1 cm2 × 10 9	< 0.001	0.321	0.181	0.571	< 0.001	0.339	0.179	0.644
Platelet count, per 103	0.061	1.005	0.999	1.010	0.381	0.381	0.179	7.159

*CLT* – clot lysis time, *NT-proBNP *– N-terminal B-type natriuretic peptide

Major bleeding and overall deaths were not associated with PC (Table 2).

## Discussion

The current study demonstrated that enhanced protein carbonylation occurs in patients with AF and may contribute to elevated risk of cerebrovascular events despite anticoagulation. This posttranslational modification was associated with a so-called prothrombotic fibrin clot phenotype, involving formation of denser fibrin fiber networks displaying reduced lysability, along with increased thrombin generation potential, which provides additional evidence for multiple prothrombotic effects related to enhanced oxidation in AF subjects. Our study expands our knowledge on causes of anticoagulant therapy failure in AF, suggesting that enhanced protein carbonylation is a potential, “prothrombotic” risk factor for a further arterial thromboembolism during long-term observation, which is hardly modifiable by the current therapeutic strategies. Measurement of circulating PC levels might help identify a subset of anticoagulated AF patients, who are prone to experience ischemic stroke and candidates to additional preventive measure to reduce this risk through strategies aimed at oxidative stress and indirectly at a prothrombotic state.

We enrolled a small, but typical AF population with similar incidence of cerebrovascular ischemic events and major bleedings during long-term follow-up as compared to large real-life trials and registries [23, 24]. Most of our patients were on DOACs, which agrees with everyday practice [25].

We have chosen PC content as a biomarker of protein oxidation which has documented relationships with fibrin clot parameters in several diseases [2–5]. The assay by Becatti et al. [5] was used in the current study. Regarding AF, to our knowledge, we presented for the first time the analysis of PC levels. Our results were comparable with those reported by authors in patients following MI and lower compared with acute stroke and hemodialysis patients as expected [5, 26]. A positive association of PC with age in our patients was consistent with other populations [4], as evidenced by several reports linking increased oxidative stress with age-related diseases [27].

Potential pathological effects of carbonylation are related to altered structure and function of numerous proteins including molecules involved in cellular energetics, metabolism regulation, membrane function and cytoskeleton composition [28]. Previous studies presented the association of protein carbonylation with thromboembolic events [3–5]. Fibrinogen as well as FV, VIII, X, XIII could be modified by posttranslational oxidative derived reactions and contribute to thromboembolic pathology [6]. In our report it was reflected by the correlation of higher PC levels with fibrin clot parameters presenting more compact fibrin network and lower potential to clot lysis, which is novel in AF subjects. Current findings are consistent with reports regarding relation of PC to prothrombotic fibrin clot phenotype in acute ischemic stroke, and diabetes [4, 29]. The reduced fibrinogen polymerization, and decreased fibrin clot potential to plasmin-dependent lysis were also correlated with plasma PC extent, and fibrinogen carbonylation in patients post MI [5]. Fibrinogen is almost 20 times more susceptible to carbonylation compared with the other plasma proteins and constituted dominant carbonylated plasma protein fraction [6, 30]. Fibrinogen carbonylation alters its function and then directly impairs clot fibrin network leading to higher clot fragility and reduces clot strength [30, 31]. Nevertheless, in subjects with aortic aneurysm, Glu1-plasminogen activation of fibrinolysis was disturbed in the mechanism of lysine side-chain carbonylation in the fibrinogen sequence [32]. Moreover, the carbonyl groups addition to plasminogen structure has been suggested as a factor impairing fibrin clot lysis what might complies with the weak correlation of PC with plasminogen activity [3]. Thus, it might be hypothesized that in AF patients, fibrinogen as well as plasminogen carbonylation could be involved in alterations to fibrin clot structure and reduced lysability.

We found increased thrombin generation expressed as ETP in AF patients with higher PC levels. Previously, PC levels did not correlate with ETP in patients with acute ischemic stroke [4]. De Cristofaro et al. [33] have postulated an imbalance between pro- and anticoagulation mechanisms as evidenced by an increase in prothrombin 1 + 2, fibrinopeptide A, and thrombomodulin due to the elevation of plasma carbonyl content in healthy volunteers. The elevated PC content might correlate with increased levels of oxidatively modified phospholipids, which could indirectly affect thrombin generation in our study [34]. Furthermore, Harutyunyan [35] demonstrated that plasma clotting rate was enhanced due to ROS exposure in the mechanism of prothrombin carbonylation. The impact of specific plasma protein carbonylation on thrombin generation in AF subjects should be evaluated in further studies.

To our knowledge, none of the studies have reported the association of PC levels with cardiovascular events during long-term follow-up [3–5]. In patients with acute ischemic stroke elevated PC levels were associated with unfavorable neurological outcomes at 3 months since the event [4]. Our intriguing observation is that PC levels at baseline in AF patients who experienced stroke during follow-up had been comparable to values reported in individuals with acute ischemic stroke [4]. This suggests a large heterogeneity of PC content in plasma in AF. The current study underlines the potential role of severe ROS exposure at baseline on further thromboembolic complications in AF patients regardless of anticoagulation [36]. Assessment of PC in AF might help identify patients at high risk of stroke on anticoagulation in whom novel therapeutic agents reducing oxidative stress with regard to protein carbonylation could offer clinical benefit, contrary to ineffective use of diet-derived antioxidants in stroke prevention [37].

Higher plasma PC in HFrEF patients with AF deserves a comment. This observation might suggest that this comorbidity specifically contributes to enhanced protein carbonylation in this clinical setting, though enhanced oxidative stress has been reported in DM, chronic kidney disease, and hypertension [8, 26, 29]. Elevated oxidative stress biomarkers were previously reported in HF [8, 13, 14]. In 27 cardiac tissue samples from HF patients, higher levels of carbonylated proteins (*P* < 0.01) were reported [38]. Despite elevated PC levels, HF patients did not display more prothrombotic state as determined by several markers in plasma. In 2010, Palka et al. [39] observed lower clot permeability in patients with HFrEF and sinus rhythm, while Matusik et al. [40] showed a tendency to longer CLT in AF patients with HF. Further studies are needed to explore the impact of HF on an oxidation-related prothrombotic state in AF patients regarding that enhanced ROS exposure is involved in thromboembolic mechanisms observed in HF [41].

The study has several limitations. Firstly, the number of patients was limited but the study was sufficiently powered to show intergroup differences and their characteristics were typical of AF patients. Secondly, patients with known potent factors altering fibrin clots parameters and thrombin generation were excluded [42], so these results cannot be extrapolated to these groups. Thirdly, laboratory investigations were performed only once, therefore changes over time cannot be excluded, though aging is related to increased carbonylation and prothrombotic markers. From a mechanistic point of view, assessment of fibrinogen carbonylation was beyond the scope of this work; the previous study by Becatti et al. [5] documented its impact on protein carbonylation. Further studies are needed to explore specific proteins affected by carbonylation and their effect of thrombin generation and fibrinolysis in AF.

In conclusion, our findings show that enhanced protein carbonylation may contribute to increased risk of thromboembolic events in AF patients despite anticoagulation and this oxidative process increases prothrombotic tendency, typical of AF, at least in part via unfavorably alterations to fibrin clot architecture and impaired fibrinolysis. Our work highlights a still poorly characterized role of protein carbonylation in thromboembolism. It might be hypothesized that attenuation of protein carbonylation might help reduced “residual” thromboembolic risk in anticoagulated AF patients.

## Supplementary Information

Below is the link to the electronic supplementary material.

Supplementary file1 (DOCX 19 KB)

## Data Availability

The data that support the findings of this study are available from corresponding author upon reasonable request.

## Abbreviations

ETP: Endogenous thrombin potential
CLT: Clot lysis time
Ks: Permeation coefficient
PC: Protein carbonylation
