# Supplementary material for: Elevated plasma protein carbonylation increases the risk of ischemic cerebrovascular events in patients with atrial fibrillation: association with a prothrombotic state
Source: J Thromb Thrombolysis. 2024 Jul 4;57(7):1206–15. doi: 10.1007/s11239-024-03003-z (PMC11496363; doi:10.1007/s11239-024-03003-z)
Supplement: Supplementary file 1 — Supplementary file1 (DOCX 19 KB) [file 11239_2024_3003_MOESM1_ESM.docx]

Online Resource 1. The sub-analysis of patients with atrial fibrillation and heart failure with reduced ejection fraction baseline characteristics.

| Variable | All patients,  N=243 | With HFrEF,  n=57 | Without HFrEF,  n=186 | P-value |
| --- | --- | --- | --- | --- |
| Age, years | 69.0 (63.0-75.0) | 60.0 (64.0-75.0) | 69.0 (62.0-74.0) | 0.44 |
| Women, n (%) | 107 (44.0) | 22 (38.6) | 107 (57.5) | 0.35 |
| BMI, kg/m^2^ | 28.0 (25.4-31.6) | 27.4 (25.7-30.9) | 28.3 (25.3-32.0) | 0.77 |
| Current smoking, n (%) | 87 (35.8) | 28 (49.1) | 59 (46.5) | 0.017 |
| LVEF, % | 49 (42-55) | 37 (36-39) | 53 (47-56) | <0.001 |
| Data on AF | | | | |
| Paroxysmal AF, n (%) | 77 (31.7) | 16 (28.1) | 61 (32.8) | 0.50 |
| Persistent AF, n (%) | 71 (29.2) | 20 (35.1) | 51 (27.4) | 0.27 |
| Permanent AF, n (%) | 95 (39.1) | 21 (36.8) | 74 (39.8) | 0.69 |
| Time from AF diagnosis, years | 6.0 (4.0-8.5) | 7.0 (4.0-8.0) | 6.0 (4.0-8.5) | 0.86 |
| CHA_2_DS_2_-VASc score, points | 4.0 (3.0-5.0) | 5.0 (4.0-6.0) | 4.0 (3.0-5.0) | <0.001 |
| Comorbidities | | | | |
| Hypertension, n (%) | 187 (77.0) | 45 (78.9) | 142 (76.3) | 0.68 |
| Diabetes mellitus, n (%) | 71 (29.2) | 19 (33.3) | 52 (30.0) | 0.44 |
| Dyslipidemia, n (%) | 213 (88.1) | 52 (91.2) | 162 (87.1) | 0.49 |
| Prior MI, n (%) | 71 (29.2) | 26 (45.6) | 45 (24.2) | 0.002 |
| Prior stroke, n (%) | 97 (39.9) | 22 (38.6) | 75 (40.3) | 0.82 |
| Medications | | | | |
| ASA, n (%) | 105 (43.2) | 29 (50.9) | 76 (40.9) | 0.18 |
| Statins, n (%) | 157 (64.6) | 38 (66.7) | 119 (64.0) | 0.71 |
| Rivaroxaban, n (%) | 80 (32.9) | 16 (28.1) | 64 (34.4) | 0.37 |
| Dabigatran, n (%) | 56 (23.0) | 9 (15.8) | 47 (25.3) | 0.14 |
| Apixaban, n (%) | 35 (14.4) | 11 (19.3) | 24 (12.9) | 0.23 |
| Warfarin, n (%) | 61 (25.1) | 16 (28.1) | 45 (24.2) | 0.56 |
| The laboratory results | | | | |
| Hemoglobin, g/dl | 13.9 (13.2-14.9) | 13.9 (13.2-14.9) | 13.8 (12.9-14.9) | 0.75 |
| White blood cell count, ×10^3^/µL | 6.7 (5.53-7.56) | 7.00 (6.05-7.96) | 6.62 (5.46-7.43) | 0.041 |
| Platelets, ×10^3^/µL | 210 (176-251) | 207 (165-277) | 210 (180-249) | 0.85 |
| Fasting glucose, mmol/L | 5.45 (4.90-6.20) | 5.50 (4.80-6.00) | 5.42 (5.00-6.22) | 0.41 |
| Creatinine, µmol/L | 82.0 (71.7-98.5) | 92.5(81.0-107.0) | 80.0 (70.7-95.0) | <0.001 |
| Total cholesterol, mmol/L | 4.53 (3.8-5.58) | 5.04 (3.97-5.61) | 4.45 (3.73-5.57) | 0.13 |
| LDL-cholesterol mmol/L | 2.59 (2.03-3.40) | 2.77 (2.29-3.52) | 2.48 (2.00-3.30) | 0.18 |
| HDL-cholesterol, mmol/L | 1.32 (1.08-1.90) | 1.32 (1.07-1.82) | 1.33 (1.14-1.63) | 0.47 |
| Triglycerides, mmol/L | 1.28 (0.85-1.76) | 1.33 (0.95-1.88) | 1.26 (0.82-1.73) | 0.13 |
| INR | 1.01 (0.95-1.07) | 1.02 (0.97-1.09) | 1.01 (0.94-1.06) | 0.28 |
| APPT, s | 29.0 (26.2-30.9) | 28.8 (26.2-30.8) | 29.1 (26.4-30.9) | 0.89 |
| NT-proBNP, pg/mL | 748 (391-1493) | 748 (450-1493) | 745 (385-1489) | 0.60 |
| Ks, ×10^−9^ cm^2^ | 6.5 (6.0-7.2) | 6.5 (5.8-7.0) | 6.6 (6.0-7.4) | 0.08 |
| CLT, min | 94 (80-108) | 99 (80-108) | 92 (80-105) | 0.35 |
| ETP, nM×min | 1502 (1427-1638) | 1497 (1425-1582) | 1535 (1428-1644) | 0.57 |
| Fibrinogen, g/L | 3.2 (2.5-3.9) | 3.5 (2.6-4.1) | 3.1 (2.5-3.8) | 0.09 |
| vWF:Ag, % | 208 (173-243) | 202 (176-259) | 208 (172-239) | 0.25 |
| TAFI:Ag, % | 100 (89-110) | 95 (87-106) | 100 (90-110) | 0.040 |
| Plasminogen, % | 105 (95-115) | 108 (96-120) | 104 (95-114) | 0.32 |
| Antiplasmin, % | 107 (96-117) | 109 (99-116) | 106 (96-117) | 0.59 |
| PAI-1:Ag, ng/mL | 14.0 (10.8-18.7) | 12.5 (9.5-16.5) | 14.4 (10.9-19.2) | 0.024 |
| Protein carbonylation, nM/mg | 3.16 (2.54-3.99) | 3.68 (2.90-4.12) | 3.00 (2.50-3.76) | 0.005 |

Values are shown as numbers (percentage) or median (interquartile range) as appropriate. Abbreviations: AF – atrial fibrillation, ASA – acetylsalicylic acid, BMI – body mass index, CRP – c-reactive protein, CLT – clot lysis time, ETP – endogenous thrombin potential, HDL-cholesterol – high-density lipoprotein cholesterol, LDL-cholesterol – low-density lipoprotein cholesterol, MI – myocardial infarction, NT-proBNP - N-terminal B-type natriuretic peptide, PAI-1:Ag - plasminogen activator inhibitor type 1 antigen, TAFI:Ag - thrombin activatable fibrinolysis inhibitor antigen, vWF:Ag – von Willebrand factor antigen
